# Liposomal bupivacaine: Is it the “code” to reduce postoperative opioid consumption?

**DOI:** 10.1097/JS9.0000000000004651

**Published:** 2026-01-07

**Authors:** Feng Yuan, Mingliang Yi, Bingbing Xiang

**Affiliations:** a Department of Anesthesiology, Chengdu Fifth People’s Hospital (The Second Clinical Medical College, Affiliated Fifth People’s Hospital of Chengdu University of Traditional Chinese Medicine), Chengdu, China; b Department of Anesthesiology, West China Hospital, Sichuan University, Chengdu, China

*Dear Editor,*

We read with considerable interest the randomized clinical trial by Ye *et al*^[1]^ investigating the efficacy of liposomal bupivacaine (intervention) versus bupivacaine hydrochloride (control) in the superficial parasternal intercostal plane (SPIP) block in cardiac surgery patients. The study’s primary outcome, cumulative opioid consumption over the first 3 postoperative days, addressed a critical clinical question, as optimizing postoperative analgesia while minimizing opioid use remains a key goal in cardiac surgical care.

The trial’s findings are noteworthy yet surprising^[1]^: while liposomal bupivacaine significantly reduced the incidence of moderate-to-severe cough-related pain within 48 h post-extubation in patients undergoing median sternotomy, it failed to reduce 3-day cumulative opioid intake compared with bupivacaine hydrochloride. This discrepancy between pain relief and opioid consumption merits in-depth analysis, as it challenges conventional expectations of regional analgesia’s role in opioid sparing and highlights important nuances in study design and pharmacologic behavior.

As the authors acknowledged, one potential explanation for this finding is the standardized use of continuous low-dose oxycodone via patient-controlled intravenous analgesia (PCIA). Such background systemic analgesia is a double-edged sword: while it ensures baseline pain control, it can also mask subtle differences in opioid requirements that might otherwise emerge between regional analgesia groups. To explore this further, a post-hoc sensitivity analysis was conducted, excluding the baseline PCIA infusion volume from the opioid consumption calculations. Notably, this analysis revealed a significant reduction in opioid use in the liposomal bupivacaine group on postoperative day 2, although no differences were observed on days 1 or 3. This time-dependent effect, which was not emphasized in the original study, aligns with the pharmacokinetic profile of liposomal bupivacaine and offers a critical context for interpreting the primary outcome.

Pharmacokinetic studies have consistently demonstrated that liposomal bupivacaine exhibits variable release kinetics in fascial and intercostal nerve blocks, characterized by an initial low-level release followed by a delayed second peak at 24–48 h post-injection^[2]^. While this prolonged release profile extends the analgesic duration compared with plain bupivacaine, the low concentration of drug released beyond 48 h is often insufficient to maintain therapeutic analgesic levels for a full 72 h. This pharmacologic property directly explains the trial’s observations: the significant reduction in pain scores at 48 h post-extubation correlates with the drug’s delayed peak effect, and the sensitivity analysis’s day-2 opioid reduction reflects this period of optimal analgesia. In contrast, bupivacaine hydrochloride exerts its peak effect within 12–24 h, accounting for equivalent opioid consumption on postoperative day 1 (when both agents are maximally effective) and waning efficacy by day 3 (when neither agent maintains sufficient analgesia, leading to similar opioid requirements).

Another important consideration omitted from the original study is the risk of rebound pain following peripheral nerve block cessation – a phenomenon recently linked to increased 30-day postoperative opioid prescriptions by Chung and colleagues^[3]^. Rebound pain occurs when the abrupt resolution of regional analgesia exposes patients to unrelieved acute pain, prompting increased opioid use. Liposomal bupivacaine’s extended analgesic duration (>48–72 h) theoretically mitigates this risk, as its gradual drug release avoids the sudden analgesic gap observed with plain bupivacaine. However, without a no-block control group, this trial could not assess this potential advantage. Including a third arm without SPIP block would have allowed for a direct comparison of opioid consumption across liposomal bupivacaine, bupivacaine hydrochloride, and no regional analgesia – providing critical data on both relative efficacy and rebound pain prevention.

Despite these limitations, the study by Ye *et al* makes a valuable contribution to the literature. The demonstration that liposomal bupivacaine reduces moderate-to-severe cough-related pain in patients undergoing cardiac surgery is clinically meaningful, as cough pain is a major source of morbidity and can impair respiratory function and wound healing. Additionally, the sensitivity analysis reinforced that liposomal bupivacaine does offer opioid-sparing effects during its peak analgesic period (postoperative day 2), even if masked by systemic background analgesia in the primary analysis.

In conclusion, this trial underscores the importance of aligning outcome measurements with the pharmacokinetic profile of regional analgesics and considering the impact of concurrent systemic analgesia on study results. Future trials evaluating liposomal bupivacaine in cardiac surgery should incorporate time-point-specific opioid consumption analyses, including no-block control groups to assess rebound pain, and optimize PCIA protocols to minimize masking of regional analgesia effects. Such design refinements will enhance our understanding of the role of liposomal bupivacaine in postoperative pain management and facilitate more informed clinical decision-making.

## Data Availability

Not applicable.
